# The Impact of Hepatitis C and Socio‐Demographic Variables on Health‐Related Quality of Life in Pakistan: Cross‐Sectional Study

**DOI:** 10.1111/jvh.70139

**Published:** 2026-02-28

**Authors:** Siwaporn Niyomsri, Aaron G. Lim, Ambreen Arif, Muhammad Asim, Auj Chaudhry, Naheed Choudhry, Aliya Hasnain, Polychronis Kemos, Chris Metcalfe, Asad Choudhry, Saeed Sadiq Hamid, Saad Niaz, Huma Qureshi, Graham R. Foster, Peter Vickerman, Josephine G. Walker, HepFreePak team, Aqsa Ramzan, Atif Saghir, Ejaz Alam, Ghayas Hai, Mohd Saadullah, Muhammad Nabeel Shafqat, Muhammad Sufyan Tahir, Taha Khan

**Affiliations:** ^1^ Bristol Medical School Population Health Sciences Bristol UK; ^2^ Doctors Plaza Karachi Sindh Pakistan; ^3^ Dow University of Health Sciences (DUHS) Karachi Sindh Pakistan; ^4^ The Liver Clinic Gujranwala Punjab Pakistan; ^5^ Queen Mary University of London London UK; ^6^ Aga Khan University Karachi Sindh Pakistan

**Keywords:** hepatitis C, Pakistan, propensity score, quality of life, quality‐adjusted life years

## Abstract

Approximately 8.8 million people are living with chronic hepatitis C virus (HCV) in Pakistan. We assessed factors related to health‐related quality of life (HRQoL) among the general population screened for HCV and calculated the national burden in quality‐adjusted life years (QALYs). A cross‐sectional study was conducted in community and clinic‐based settings in Karachi and Gujranwala. HRQoL was assessed before diagnosis using EQ‐5D‐3L (Pakistan value set). Propensity score matching (PSM) was used to address socio‐economic differences between HCV RNA‐positive (viraemic) and HCV‐antibody‐negative participants. We assessed socio‐demographic and HCV‐related predictors of HRQoL (Tobit regression) and problems by EQ‐5D domain (logistic regression). The HCV transmission model was used to estimate the burden of HCV in terms of morbidity‐ and mortality‐related QALY loss in 2024. After PSM, 778 individuals remained in each group from a total of 5468 participants. HCV‐positive participants had lower HRQoL (EQ‐5D‐3L score, *p* < 0.001) and higher odds of problems in all five EQ‐5D dimensions. Lower HRQoL was associated with older age and unemployment, while married or Urdu‐speaking participants had higher HRQoL. There was little evidence that cirrhosis was associated with HRQoL (*p* = 0.140) among HCV‐positive participants. The total estimated QALY loss due to HCV in Pakistan in 2024 was 804,580 QALYs, of which 55% was due to mortality. HCV infection is associated with reduced HRQoL and substantial QALY losses in Pakistan. Our findings emphasise the role of socio‐demographic variables on HRQoL. Further research in Pakistan is needed to determine if HCV treatment can mitigate these effects.

## 1 Introduction

Globally, approximately 50 million people are infected with hepatitis C virus, with 1 million new infections occurring each year, a third of which occur in Pakistan [1]. Although there is no vaccine against HCV, pan‐genotypic direct‐acting antivirals (DAAs) are oral regimens taken for 12–24 weeks that cure 95% of HCV infections. Global targets for the elimination of viral hepatitis as a public health threat aim to reduce new HCV infections by 80% and deaths by 65% compared to 2015 [1]. Pakistan has one of the highest global burdens of HCV, with an estimated viraemic prevalence of 4.3%, or 8.8 million people infected in 2021, representing nearly 18% of the global burden of HCV [1, 2, 3, 4].

Patient‐reported outcomes, such as health‐related quality of life (HRQoL), offer insights into how patients perceive their illness and treatment, including physical and psycho‐social dimensions beyond clinical endpoints [5]. HRQoL measures are widely used in cost‐effectiveness analyses through conversion to quality‐adjusted life years (QALYs) [6].

Previous studies in high‐income countries have demonstrated that HCV infection is associated with a decline in HRQoL [7, 8, 9, 10], but there is limited evidence on the impact in Pakistan or other low‐ and middle‐income countries [11, 12, 13, 14]. In Pakistan, HCV infection is mostly transmitted through unsafe medical practices, such as the reuse of syringes and contaminated medical equipment. Additionally, genotype 3 is most common in Pakistan, accounting for 80% of cases, and leads to faster disease progression than other genotypes that are prevalent globally [15, 16]. These epidemiological differences, along with differences in socio‐economic and healthcare disparities, indicate the importance of context‐specific research on the impact of HCV on HRQoL and the burden of disease in Pakistan.

The few previous studies in Pakistan indicate a detrimental impact of HCV infection on patients' well‐being and HRQoL, but are limited in scope and generalisability [11, 12]. One study used the EQ‐5D 3‐Level Version (EQ‐5D‐3L) to assess HRQoL in HCV patients receiving pharmacological therapy in rural Punjab, finding that 60% of patients experienced a marked reduction in HRQoL. However, this study did not convert EQ‐5D‐3L results into valued utility weights, and the findings cannot be generalised to the broader Pakistani population due to its small sample size and focus on a single village [11]. Another study in the Sargodha district used the World Health Organization Quality of Life‐BREF (WHOQOL‐BREF) tool to evaluate HRQoL in HCV and HBV patients, finding that HCV patients had a lower HRQoL than patients with HBV [12].

In this study, we evaluate HRQoL of the Pakistani population with and without HCV using EQ‐5D‐3L, in order to identify drivers of HRQoL among people with HCV (with and without cirrhosis) and to compare their HRQoL to people without HCV. In addition, we use the recently published EQ‐5D value set for Pakistan to compare utility weights among different population subgroups and apply them in a previously published model of HCV transmission in Pakistan to estimate the QALY losses due to HCV for Pakistan [17, 18].

## 2 Methods

### 2.1 Patient Recruitment

This study was part of the HepFREEPak programme, which aims to measure HCV treatment efficacy in Pakistan, examine the impact of an expanded access test and treat programme and determine cost‐effective approaches to eliminate HCV in Pakistan. A cross‐sectional study was conducted in clinic and community‐based treatment units in Karachi and Gujranwala, Pakistan, as part of the enrolment and screening phase of a prospective cohort study from November 2021 to August 2023, with data accessed in October 2024.

Participants were recruited primarily through random household screening or organised screening camps in communities and at workplaces (see S1). Individuals aged 18 years and older who consented to undergo hepatitis C testing were eligible unless they met the following exclusion criteria: prior history of chronic HCV; clinically significant illnesses such as advanced liver disease, severe renal disease, severe cardiovascular diseases, or severe diabetes with complications; and comorbidities with a life expectancy of less than 12 months. Determination of HCV infection was based on positive results for both anti‐HCV antibody and HCV RNA tests. All individuals who tested positive for both tests were denoted as HCV positive and having active infection (RNA positive; defined as HCV RNA detectable with a molecular diagnostic assay with a sensitivity of > 100 IU/mL). Individuals who were anti‐HCV antibody‐positive but HCV RNA‐negative (either from self‐clearance of the virus or prior treatment) were excluded from both arms as they did not have active infection. The HCV‐negative cohort was based on anti‐HCV antibody‐negative at the screening. Cirrhosis diagnosis was determined among HCV‐positive patients based on the presence of clinical features (ascites/past or current decompensation) or evidence of liver cirrhosis according to APRI (> 1.5) or level of liver stiffness by FibroScan (> 12.5 kPa) or ultrasound features of cirrhosis. Patients without cirrhosis were treated with DAA‐based medications (sofosbuvir/daclatasvir) for 12 weeks, while patients with cirrhosis received 24 weeks of treatment with or without ribavirin, in accordance with clinical guidelines; treatment duration as reported in the dataset was used as a proxy for cirrhosis status.

### 2.2 Socio‐Demographic Data and HRQoL

To minimise the potential labelling effect, subjects completed an interviewer‐administered survey (in Urdu) before the results of the HCV antibody test were given to the individual or the health care professional performing the test. The survey included questions on socio‐demographic characteristics, including study site, age, gender, marital status, employment status, area of residence, ethnicity and education levels.

To evaluate a patient's HRQoL, the survey included the EQ‐5D‐3L questionnaire (Urdu translation) [6]. The EQ‐5D‐3L questionnaire assesses individuals across five domains: mobility, self‐care, usual activities, pain/discomfort and anxiety/depression, with three levels included for each domain: no problems, moderate problems or severe problems. Additionally, participants rate their current overall health status on a visual analogue scale (VAS) ranging from 0 (worst imaginable health) to 100 (best imaginable health). The five‐dimensional scores were converted into a single HRQoL index (QALY weight) ranging from 0 to 1, using the value set for Pakistan [17]. Higher scores indicate a better quality of life, while values below 0 indicate health states worse than death (1 = full health; 0 = dead).

### 2.3 Propensity Score Matching (PSM) and Regression Analysis

Socio‐economic deprivation is associated with a higher incidence of HCV, and consequently, estimates of the causal effect of HCV status on HRQoL are subject to confounding by socio‐economic factors [19]. To address this issue, propensity score matching (PSM) was conducted to reduce selection bias and strengthen the validity of causal inferences by creating comparable groups balanced on socio‐demographic characteristics.

The PSM dataset was then used for regression analyses to assess differences in HRQoL between HCV status. Given the censored nature of QALY scores, with a large proportion of participants having a score of 1, multivariable Tobit regression analysis was used to investigate the effects of HCV status and other characteristics on overall HRQoL (EQ‐5D score). We fit Tobit models with right‐censoring only, which permits valid negative EQ‐5D values. A negative coefficient (B) means that the variable is associated with lower HRQoL, while a positive coefficient indicates higher HRQoL. The magnitude of the coefficient shows how much EQ‐5D score is expected to change.

VAS scores were assessed using standard linear regression. Problems in each EQ‐5D dimension were evaluated using logistic regression with a binary outcome for reporting either moderate or severe problems compared to no problems, by HCV status and other characteristics. In addition, Tobit regression analysis was also used to assess differences in HRQoL between HCV‐positive individuals with and without suspected cirrhosis. This analysis used the full prematching dataset (*n* = 998) because it focused only on variation within the HCV‐positive group.

To assess model robustness and consistency, sensitivity analyses were performed on the PSM‐matched sample using linear and logistic regression for EQ‐5D < 1; see S3. All analyses were done using R version 4.3.1 using tableone, censReg and MatchIt packages [20, 21, 22, 23].

### 2.4 QALY Burden of HCV in Pakistan

We estimated the overall QALY losses due to HCV in Pakistan for 2024 using projected case numbers per year drawn from a previously published dynamic HCV transmission and disease progression model for Pakistan [18]. To estimate morbidity‐related QALY losses, we multiplied stage‐specific disutilities by the number of cases in each stage (noncirrhosis, compensated cirrhosis, decompensated cirrhosis and HCC), based on our study results and literature values for decompensated cirrhosis and HCC [24, 25]. Mortality‐related QALY loss was based on people who died prematurely from HCV‐related causes and would otherwise still be alive in 2024, as estimated by the model. The cumulative years of life lost (YLL) were calculated using the average life expectancy in Pakistan (68 years) and the baseline HRQoL of HCV‐negative patients to estimate the QALY loss [26]. Total QALY loss was the sum of morbidity‐ and mortality‐related losses (see S2 for details).

## 3 Results

### 3.1 Participant Characteristics and Propensity Score Matching Results

Table 1 presents the socio‐demographic characteristics before and after PSM. Of the total participants, the median age of participants was 39 years (interquartile range [IQR] 30–50), and 59% were male. The majority of participants were recruited from Karachi (84%), Pakistan's largest city and the primary study site. Among the study subjects, there were 1263 (23%) HCV‐positive individuals (anti‐HCV antibody and HCV RNA positive), with 51% of these cases occurring among individuals aged 40–59 years. A large proportion of HCV‐positive individuals had no formal education (65% vs. 27%), were unemployed (60% vs. 24%), and resided in rural areas (84% vs. 23%) when compared with HCV‐negative individuals (Table 1).

**TABLE 1:** Socio‐demographic characteristics of study participants before and after propensity score matching.

Characteristics	Before matching (*N* = 5468)						After matching (*N* = 1556)					
	HCV negatives		HCV positives		SMD*	*p* **	HCV negatives		HCV positives		SMD*	*p* **
	*N*	%	*N*	%			*N*	%	*N*	%		
Gender												
Male	2712	64.49	522	41.33	0.48	< 0.001	376	48.33	350	44.99	0.07	0.2
Female	1493	35.51	741	58.67			402	51.67	428	55.01		
Age												
≤ 29	1224	29.11	125	9.9	0.72	< 0.001	121	15.55	120	15.42	0.03	0.99
30–39	1227	29.18	232	18.37			192	24.68	191	24.55		
40–49	930	22.12	343	27.16			211	27.12	204	26.22		
50–59	530	12.6	296	23.44			154	19.79	158	20.31		
60 and over	294	6.99	267	21.14			100	12.85	105	13.5		
Marital status												
Single	861	20.48	76	6.02	0.51	< 0.001	71	9.13	66	8.48	0.03	0.86
Married	3249	77.27	1075	85.11			670	86.12	672	86.38		
Divorced/widowed	92	2.19	112	8.87			37	4.76	40	5.14		
Employment status												
Employed	3201	76.12	506	40.06	0.79	< 0.001	447	57.46	446	57.33	< 0.01	1
Unemployed	1004	23.88	757	59.94			331	42.54	332	42.67		
Residential area												
Urban	3234	76.91	200	15.84	1.55	< 0.001	221	28.41	200	25.71	0.06	0.25
Rural	971	23.09	1063	84.16			557	71.59	578	74.29		
Study site												
Karachi	3388	80.57	1196	94.7	0.44	< 0.001	682	87.66	712	91.52	0.13	0.02
Gujranwala	817	19.43	67	5.3			96	12.34	66	8.48		
Ethnicity												
Punjabi	1030	24.49	106	8.39	1.26	< 0.001	111	14.27	133	17.1	0.15	0.08
Sindhi	970	23.07	633	50.12			121	15.55	99	12.72		
Baluch	242	5.76	333	26.37			136	17.48	162	20.82		
Urdu‐speaking	1350	32.1	32	2.53			384	49.36	352	45.24		
Other	613	14.58	159	12.59			26	3.34	32	4.11		
Education												
No education	1128	26.83	822	65.08	0.95	< 0.001	430	55.27	432	55.53	0.02	0.98
Primary school	848	20.17	218	17.26			153	19.67	157	20.18		
High school	932	22.16	157	12.43			132	16.97	127	16.32		
University, college, and others	1297	30.84	66	5.23			63	8.1	62	7.97		

*Note:* HCV Positive: Participants with positive results for both anti‐HCV antibody and HCV RNA tests. HCV Negative: Participants with negative result for anti‐HCV antibody.

Abbreviations: HCV, hepatitis C virus; SMD, standardised mean difference.

* SMD < 0.1 are considered to indicate good balance.

** *p* values before matching were calculated using Chi‐square tests comparing between HCV‐negative and HCV‐positive groups. After matching, *p* values were based on McNemar or McNemar–Bowker tests.

After PSM, 778 HCV‐positive individuals were matched to 778 HCV‐negative participants based on age, gender, marital status, education level, residential areas, ethnicity, study site and employment status. Postmatching, the SMD for propensity score distance was reduced from 1.69 before matching to 0.09 after matching, with a mean propensity score for each arm after matching of 0.483, indicating that the groups were well‐matched on the observed covariates. SMDs were below 0.1 for nearly all covariates and there was no longer evidence of a difference in the distribution of covariates between HCV‐positive and ‐negative groups (*p* > 0.05), indicating the matching process effectively balanced the groups. Detailed characteristics of the matched participants are provided in Table 1 and Figure S1.

### 3.2 EQ5D and VAS Scores

Prior to PSM, EQ‐5D and VAS scores displayed left‐skewed distributions with clustering of scores at the upper end of the scale (1.0 for EQ‐5D). HCV‐negative participants scored higher on both measures compared to HCV‐positive participants (Table 2; Figure S2 in the S4). Regression models, adjusted by including measures of age, gender, education, residential area, study site, employment, marital status and ethnicity as covariates, indicated that these differences in scores were unlikely to have arisen by chance (*p* < 0.001).

**TABLE 2:** EQ5D and VAS results between HCV‐negative and HCV‐positive groups.

Outcomes	Estimates by group			Adjusted effect of HCV status on HRQoL*	
	HCV‐negative	HCV‐positive	*p* (crude comparison)**	Estimate (95% CI)	*p*
Before PSM *n* EQ‐5D VAS	Mean (SD) 4205 0.979 (0.059) 80.20 (13.68)	Mean (SD) 1263 0.952 (0.081) 67.86 (13.87)	< 0.001 < 0.001	B (95% CI) Tobit: −0.06 (−0.07, −0.04) Linear: −5.63 (−6.69, −4.57)	< 0.001 < 0.001
After PSM *n* EQ‐5D VAS	Mean (SD) 778 0.971 (0.069) 75.90 (13.81)	Mean (SD) 778 0.960 (0.071) 69.77 (14.27)	< 0.001 < 0.001	B (95% CI) Tobit: −0.05 (−0.07, −0.04) Linear: −5.86 (−7.22, −4.51)	< 0.001 < 0.001
EQ‐5D Dimension (moderate to severe problems) After PSM Mobility difficulties Usual activities difficulties Pain/discomfort Self‐care difficulties Anxiety/depression	% 14.01 8.48 16.20 5.91 16.58	% 25.71 10.41 20.57 10.03 19.54	< 0.001 0.199 0.024 0.003 0.165	Logistic, OR (95% CI) 2.38 (1.80, 3.16) 1.53 (1.04, 2.25) 1.69 (1.25, 2.28) 2.37 (1.54, 3.66) 1.32 (1.00, 1.75)	< 0.001 0.030 0.001 < 0.001 0.048

Abbreviations: 95% CI, 95% confidence interval; B, beta estimates; EQ5D, European Quality of Life 5 Dimensions; HCV, hepatitis C virus; PSM, propensity score matching; VAS, Visual Analog Scale.

* Regression results estimating the effect of HCV status on EQ‐5D and VAS scores using Tobit (for censored EQ‐5D index) and linear models (for VAS), adjusted for age, gender, education, residential area, study site, employment, marital status and ethnicity. Details of full regression models are shown in the Supporting Information.

** *p* values in the descriptive columns (left) were from crude (unadjusted) comparison; calculated using Wilcoxon rank‐sum tests before PSM and Wilcoxon signed‐rank tests after PSM.

After further adjusting for demographic differences through PSM, there remained strong evidence that HCV‐negative participants had higher mean EQ‐5D (0.971 ± 0.069) and VAS scores (75.90 ± 13.81) than HCV‐positive participants (EQ‐5D: 0.960 ± 0.071; VAS: 69.77 ± 14.27), (Table 2). Full results by socio‐demographic subgroup are provided in Table S5. Both a crude comparison between the PSM groups (*p* < 0.001) and the comparison adjusting for socio‐demographic measures (B = −0.05, 95% CI: −0.07 to −0.04, *p* < 0.001) indicated strong evidence that these differences were unlikely to have arisen by chance (Tables 2 and S6). A coefficient of −0.05 suggests that, on average, HCV‐positive participants had EQ‐5D scores 0.05 points lower than HCV‐negative, which is equivalent to about 5% of the full EQ‐5D scale. These findings were consistent in sensitivity analyses on linear and logistic models, supporting robustness (see Table S3).

Among HCV‐positive participants in the PSM analysis set (*n* = 998), further analysis was conducted to explore associations with HRQoL (Table 3). There was evidence that older age was associated with lower EQ‐5D scores. Compared with those aged ≤ 29, older ages had progressively lower scores (all *p* < 0.001). There was also evidence of lower EQ‐5D scores among participants who were unemployed (*p* = 0.002), and single or divorced/widowed (*p* = 0.004). Living in Karachi (compared to Gujranwala) was associated with higher EQ‐5D scores (*p* = 0.001). There was no strong evidence that gender, residential area, ethnicity and education level were associated with EQ‐5D scores in the HCV‐positive group.

**TABLE 3:** Tobit regression analysis of characteristics related to EQ‐5D scores in all HCV‐positive participants (*n* = 998).

Parameters	B	SE	95% CI	*p*
Intercept	1.01	0.04	0.94, 1.08	< 0.001
Age [ref: ≤ 29]				
30–39	−0.07	0.02	−0.11, −0.02	< 0.001
40–49	−0.08	0.02	−0.13, −0.04	
50–59	−0.09	0.02	−0.13, −0.05	
60 and over	−0.13	0.02	−0.17, −0.08	
Study site [ref: Gujranwala]				
Karachi	0.12	0.04	0.04, 0.19	0.001
Gender [ref: female]				
Male	0.01	0.01	−0.01, 0.04	0.259
Residential area [ref: rural]				
Urban	0.03	0.02	−0.01, 0.07	0.059
Employment status [ref: employed]				
Unemployed	−0.04	0.01	−0.06, −0.01	0.002
Marital status [ref: married]				
Single	−0.05	0.02	−0.10, −0.01	0.004
Divorced or widowed	−0.05	0.02	−0.08, −0.01	
Ethnicity [ref: Punjabi]				
Urdu‐speaking	0.06	0.04	−0.02, 0.15	0.16
Baluch	−0.02	0.03	−0.07, 0.04	
Sindhi	0.01	0.03	−0.05, 0.06	
Other	0.02	0.03	−0.04, 0.08	
Education level [ref: primary school]				
No education	−0.01	0.02	−0.04, 0.02	0.114
Secondary school	0.04	0.02	−0.01, 0.08	
University and others	−0.01	0.03	−0.06, 0.05	
Treatment duration [ref: 12‐week]				
24‐week	−0.02	0.01	−0.04, 0.01	0.14
LogSigma	−1.97	0.04	−2.05, −1.90	< 0.001

Abbreviations: B, beta estimates; CI, confidence interval; ref, reference; SE, standard error.

To assess the relationship between cirrhosis and EQ‐5D, treatment duration was used as a proxy for suspected cirrhosis. There was little evidence that participants with 24‐week treatment duration reported lower EQ‐5D scores than those participants undergoing 12‐week treatment (B = −0.02, 95% CI: −0.04 to 0.01, *p* = 0.140; Table 3 and S7 for descriptive outcomes).

### 3.3 Comparison of EQ‐5D Dimensions

In the PSM dataset, the HCV‐positive group reported a higher prevalence of moderate‐to‐severe problems across all 5 EQ‐5D dimensions compared to the HCV‐negative group (Table 2). Descriptively, the largest differences were observed in mobility (25.7% vs. 14.0%) and self‐care difficulties (10.0% vs. 5.9%). Logistic regression models adjusting for socio‐demographic variables confirmed evidence of a higher odds of moderate or severe problems for all domains compared to the HCV‐negative group (Tables 2 and S8).

Among HCV‐positive individuals (using full HCV‐positive dataset), older age and unemployment were consistently associated with more health problems across EQ‐5D domains (Table 4). There was evidence that gender, marital status and ethnicity were associated with specific domains, while duration of treatment (indicating cirrhotic disease) was not associated with any of the EQ‐5D domains.

**TABLE 4:** Logistic regression analysis results for variables associated with problems in EQ‐5D domains among HCV‐positive participants.

Independent variables	Mobility difficulties		Usual activities difficulties		Pain/discomfort		Self‐care difficulties		Anxiety/depression	
	OR (95% CI)	*p*	OR (95% CI)	*p*	OR (95% CI)	*p*	OR (95% CI)	*p*	OR (95% CI)	*p*
Intercept	0.36 (0.12–1.07)	0.067	0.04 (0.01–0.27)	0.001	1.17 (0.38–3.59)	0.788	0.09 (0.01–0.53)	0.008	0.24 (0.08–0.69)	0.008
Study site [ref: Gujranwala]										
Karachi	0.55 (0.19–1.57)	0.267	0.27 (0.08–0.90)	0.032	0.08 (0.03–0.25)	< 0.001	0.21 (0.05–0.87)	0.031	0.72 (0.27–1.88)	0.498
Age [ref: ≤ 29]										
30–39	2.07 (1.06–4.03)	0.033	6.23 (1.38–28.05)	0.017	1.59 (0.80–3.14)	0.184	6.21 (1.37–28.03)	0.018	1.77 (0.90–3.49)	0.099
40–49	2.85 (1.51–5.37)	0.001	7.18 (1.64–31.46)	0.009	1.80 (0.94–3.45)	0.076	6.81 (1.56–29.80)	0.011	2.16 (1.13–4.15)	0.02
50–59	3.14 (1.63–6.04)	< 0.001	9.17 (2.06–40.78)	0.004	2.23 (1.15–4.35)	0.018	11.93 (2.69–52.83)	0.001	2.16 (1.10–4.24)	0.026
60 and over	7.25 (3.73–14.07)	< 0.001	16.80 (3.80–74.29)	< 0.001	2.85 (1.45–5.59)	0.002	18.08 (4.07–80.30)	< 0.001	2.31 (1.16–4.61)	0.018
Gender [ref: female]										
Male	0.49 (0.35–0.68)	< 0.001	0.92 (0.59–1.43)	0.71	1.04 (0.74–1.47)	0.82	0.44 (0.28–0.71)	0.001	1.09 (0.77–1.54)	0.64
Residential areas [ref: rural]										
Urban	0.42 (0.24–0.73)	0.002	0.94 (0.45–1.93)	0.856	0.75 (0.43–1.32)	0.321	1.55 (0.24–1.23)	0.145	1.10 (0.68–1.79)	0.704
Ethnicity [ref: Punjabi]										
Urdu‐speaking	0.42 (0.11–1.56)	0.193	0.22 (0.03–1.97)	0.178	0.09 (0.01–0.79)	0.029	0.32 (0.03–3.11)	0.325	0.18 (0.05–0.69)	0.013
Marital status [ref: married]										
Single	0.91 (0.44–1.88)	0.798	2.46 (0.98–6.21)	0.056	1.48 (0.73–3.02)	0.28	2.91 (1.20–7.06)	0.018	1.18 (0.58–2.39)	0.648
Divorced or widowed	1.52 (0.95–2.43)	0.084	1.04 (0.58–1.86)	0.907	1.20 (0.73–1.97)	0.47	1.25 (0.71–2.19)	0.436	2.01 (1.24–3.26)	0.005
Employment [ref: employed]										
Unemployed	1.36 (1.00–1.85)	0.05	1.91 (1.22–2.99)	0.005	1.90 (1.34–2.68)	< 0.001	1.08 (0.71–1.64)	0.73	1.70 (1.20–2.39)	0.003

Abbreviations: 95% CI, 95% confidence interval; HCV, hepatitis C virus; OR, odds ratio; *p*, *p* value; ref., reference.

### 3.4 QALY Burden of HCV in Pakistan

In 2024, morbidity‐related QALY loss due to HCV infection in Pakistan was estimated at 362,300 QALYs, or 147.75 per 100,000 person‐years. Noncirrhosis HCV infection contributed the most to this loss, accounting for 162,158 QALYs (45% of the total morbidity‐related QALY loss, or 66.13 per 100,000 person‐years), primarily due to the large number of infections (Table 5). The mortality‐related QALY loss due to HCV infection was estimated at 442,280 QALYs or 180.37 per 100,000 person‐years, indicating the impact of HCV infection on premature deaths. When combining morbidity and mortality burdens, the total QALY loss of HCV infection in Pakistan, accounting for both morbidity and mortality, was estimated at 804,580 QALYs per year, with an uncertainty range of 489,562–1,243,416 QALYs.

**TABLE 5:** Annual QALY losses due to HCV in Pakistan (2024).

Component	QALY loss	Uncertainty range
Noncirrhosis HCV	162,158	104,817–224,660
Compensated cirrhosis	55,447	20,945–106,661
Decompensated cirrhosis	118,856	51,127–208,517
Hepatocellular carcinoma	25,840	2901–90,316
Morbidity QALY loss*	362,300	179,790–630,153
Mortality QALY loss**	442,280	390,773–613,263
Total QALY loss	804,580	489,562–1,243,416

Abbreviations: HCV, hepatitis C virus; QALY, quality‐adjusted life year.

* Morbidity losses reflect stage‐specific disability weights and represent the sum of QALY losses across all disease stages.

** Mortality losses were based on premature death estimates. Full methodological details and population inputs are provided in S2.

## 4 Discussion

This study is the first large‐sample, multi‐centre investigation of HRQoL in Pakistani communities and clinic‐based settings, providing critical insights into the population‐level impact of HCV infection. Understanding HRQoL of affected individuals is essential for informing public health interventions and improving patient outcomes in this high burden setting [27]. Our findings reveal that HCV‐negative participants consistently reported higher HRQoL than HCV‐positive participants across both EQ‐5D and VAS scores. These differences remained after adjusting for socio‐demographic factors such as age, gender, employment, ethnicity, urbanicity and marital status, factors that are themselves associated with both HCV infection and HRQoL. There was little evidence of a difference in HRQoL between individuals with suspected cirrhosis (24‐week treatment group) and those without suspected cirrhosis (12‐week treatment group), as measured by EQ‐5D scores.

### 4.1 Strengths and Limitations

Strengths of our study include that it is the first large‐scale study in Pakistan to estimate utility values for HCV patients using the EQ‐5D tool and to apply the recently published EQ‐5D Pakistani value set, providing more accurate HRQoL values for the local population [17]. While we found that factors such as older age, female gender, unemployment and being single or divorced/widowed were significantly correlated with a lower HRQoL among HCV‐positive individuals, these patterns are consistent with previous findings in other population settings. Furthermore, we estimate the total burden of HCV using a dynamic model and accounting for disutility weights within noncirrhotic HCV patients, which are overlooked in DALY burden estimates.

A key limitation of our study is that we lacked information on clinical liver disease stages, so we used treatment duration as a proxy for cirrhosis. This limited our ability to detect differences due to liver disease among HCV‐positive individuals or to attribute the reduced HRQoL among people with HCV to liver disease. Our study did not include other comorbidities such as diabetes, chronic kidney disease (CKD), or HIV infection that could co‐occur with HCV and may impact HRQoL; therefore, it is difficult to determine whether HRQoL as observed represents HCV alone or is combined with other chronic conditions.

We did not include individuals with anti‐HCV positive and RNA negative or posttreatment follow‐up. Findings in this study reflect baseline HRQoL during active viraemia. In addition, the cross‐sectional design limits causal inference; although PSM aims to reduce bias, reverse causality and residual confounding may remain after covariate adjustment.

### 4.2 Comparison With Other Studies

Our study suggests that 10%–26% of HCV‐positive patients experience moderate to severe problems in each EQ‐5D dimension, with problems with mobility, pain/discomfort and self‐care most reported. A previous study in Pakistan using the EQ‐5D‐5 L questionnaire found that around 35% of HCV‐positive patients had severe problems with walking, 34% had severe difficulties performing their usual activities and 47% experienced extreme pain; the higher reported problems were likely due to more advanced liver disease and side effects of interferon‐based treatment [11]. The impact of HCV on HRQoL is supported by a qualitative study on personal experiences of HCV infection in South Punjab, Pakistan, which showed that many participants reported bodily pains, malaise and lethargy, although some showed no clinically significant symptoms before diagnosis [28]. Similarly, studies from outside Pakistan have identified that HCV patients often experience nonspecific extra‐hepatic symptoms that impact physical well‐being, emotional problems, physical functioning and general health perceptions [13, 29], as well as high rates of depression [30].

Our study reported the mean EQ5D score as 0.979 for the HCV‐negative group and 0.952 for the HCV‐positive group. This is higher than other studies, a systematic literature review and meta‐analysis concluded that among Asian populations, the EQ5D values for genotype 1 HCV‐infected patients without cirrhosis ranged from 0.871 to 0.940 [24]. The high QALY values reported in our study compared to other studies may reflect a difference in the value set for Pakistan, reflecting the influence of local value preferences [31], we have investigated this by sensitivity analysis using the UK value set, which results in 0.920 for the HCV‐negative group and 0.888 for the HCV‐positive group (see S5).

Our findings related to socio‐demographic drivers of HRQoL in HCV align with other studies. Gender affected HRQoL, with women with HCV‐positive experiencing substantially lower HRQoL compared to men; in a study in China, women with HCV reported more problems in pain or discomfort and anxiety or depression compared to men [14], while in Pakistan, female participants with HCV exhibited a greater impact of illness on their emotional well‐being and HRQoL [32]. Age also exhibited a strong negative impact on HRQoL in HCV patients in our study, consistent with previous studies indicating that older age was significantly associated with impaired physical HRQoL [33, 34]. Unemployment has been found to be significantly correlated with decreased HRQoL in several studies of HCV [35, 36, 37]. Previous studies have found differences in HRQoL based on education, which we did not observe in this study [31, 38].

The total QALY burden estimated in our study (804,580 QALYs) is comparable to the 765,713 DALYs (562,512–1,016,325) reported by the GBD 2021 for hepatitis C in Pakistan [39]. However, our study estimate of morbidity‐related QALY loss (362,300 QALYs) was much higher than the 7214 years of life with disability (YLDs) estimated by GBD. This discrepancy is largely explained by how early stages of HCV are incorporated in each framework [26]. The GBD framework assigns a disability weight of 0 to all states of HCV less severe than decompensated cirrhosis, meaning that in earlier stages of infection or in patients without liver failure, HCV is not considered to cause disability (Table S4), leading to a low morbidity‐related burden of disease. In our estimate, noncirrhotic HCV accounted for the largest share of the morbidity burden due to its high prevalence. As we saw in our analysis, HCV without cirrhosis is likely to be associated with physical symptoms that negatively affect the overall quality of life [8, 40, 41].

## 5 Conclusions

The burden of HCV in Pakistan impacts HRQoL beyond hepatic symptoms. The overall HRQoL burden of HCV in Pakistan is high, especially among underserved groups. While national elimination efforts are ongoing, gaps remain in screening and treatment access. These findings emphasise the importance of scaling up existing interventions and applying a more targeted approach to reduce the broader impact of HCV infection.

## HepFreePak team Consortium Authors

Aqsa Ramzan, Atif Saghir, Ejaz Alam, Ghayas Hai, Mohd Saadullah, Muhammad Nabeel Shafqat, Muhammad Sufyan Tahir, Taha Khan.

## Author Contributions

Siwaporn Niyomsri: conceptualisation, methodology, formal analysis, writing, original draft. Aaron G. Lim: methodology, software, writing, review and editing. Ambreen Arif, Muhammad Asim, Auj Chaudhry, Aliya Hasnain: investigation. Naheed Choudhry: investigation, data curation, writing, review and editing, project management. Polychronis Kemos: database design. Chris Metcalfe, Peter Vickerman: conceptualisation, writing, review and editing. Graham .R. Foster: resources, writing, review and editing, funding acquisition. Asad Choudhry, Huma Qureshi: investigation, resources. SaN, Saeed Sadiq Hamid: investigation, resources, funding acquisition. Josephine G. Walker: conceptualisation, methodology, writing, review and editing, supervision.

## Funding

This project was funded by the Wellcome Trust, ‘Treating hepatitis C in Pakistan. Strategies to avoid resistance to antiviral drugs’ Code: 220866/Z/20/Z. This work was supported by the University of Bristol's International Science Partnerships Fund (ISPF) Institutional Support Grant (ODA), funding provided by Research England (grant reference RE‐CL‐2023‐09).

## Conflicts of Interest

Josephine G. Walker and Peter Vickerman have received investigator‐sponsored research grants from Gilead Sciences, unrelated to this work. All other authors declare no conflicts of interest.

## Supporting information


**Table S1:** DC and HCC HRQoL taken from previously studies.
**Table S2:** Disutilities used in the QALY loss analyses; baseline HRQoL value for individuals without HCV was derived from the HCV‐negative group, with a mean EQ5D score of 0.979 (0.977–0.981).
**Table S3:** Numbers of HCV infection, deaths and YLLs modelled as of 2024.
**Table S4:** GBD—DALY and QALY weight comparison.
**Figure S1:** The distribution of propensity scores (top) and covariance balance plot before and after propensity matching for HCV‐positive and ‐negative groups (bottom).
**Table S5:** Sensitivity analyses results on matched sample.
**Table S6:** QuEENS Checklist (TSD‐17).
**Figure S2a:** Scatter plot of EQ5D and VAS scores of all participants.
**Figure S2b:** Distribution of EQ5D and VAS scores of all participants.
**Table S7:** EQ5D and VAS results by socio‐demographic characteristics among HCV‐positive and HCV‐negative after propensity score matching.
**Table S8:** Tobit regression results showing the relationship between HCV‐negative and HCV‐positive among all participants using propensity‐score matched data.
**Table S9:** EQ5D and VAS results in HCV positives by treatment duration (proxy for cirrhosis status, with cirrhosis patients treated for 24 weeks), for all participants without propensity score matching; EQ‐5D HRQoL weights calculated using Pakistan value set.
**Table S10:** Logistic regression results for EQ5D domains between HCV‐negative and HCV‐positive after PSM.
**Table S11:** Full logistic regression results for variables associated with problems in EQ‐5D domains among HCV‐positive participants.
**Table S12:** EQ5D results comparing HCV‐positive and HCV‐negative groups using the UK value set (matched data).

## Data Availability

Research data are not shared.
