# Supplementary material for: The Impact of Hepatitis C and Socio‐Demographic Variables on Health‐Related Quality of Life in Pakistan: Cross‐Sectional Study
Source: J Viral Hepat. 2026 Feb 28;33(4):e70139. doi: 10.1111/jvh.70139 (PMC12949456; doi:10.1111/jvh.70139)
Supplement: Supplementary file 1 — Table S1: DC and HCC HRQoL taken from previously studies. Table S2: Disutilities used in the QALY loss analyses; baseline HRQoL value for individuals without HCV was derived from the HCV‐negative group, with a mean EQ5D score of 0.979 (0.977–0.981). Table S3: Numbers of HCV infection, deaths and YLLs modelled as of 2024. Table S4: GBD—DALY and QALY weight comparison. Figure S1: The distribution of propensity scores (top) and covariance balance plot before and after propensity matching for HCV‐positive and ‐negative groups (bottom). Table S5: Sensitivity analyses results on matched sample. Table S6: QuEENS Checklist (TSD‐17). Figure S2a: Scatter plot of EQ5D and VAS scores of all participants. Figure S2b: Distribution of EQ5D and VAS scores of all participants. Table S7: EQ5D and VAS results by socio‐demographic characteristics among HCV‐positive and HCV‐negative after propensity score matching. Table S8: Tobit regression results showing the relationship between HCV‐negative and HCV‐positive among all participants using propensity‐score matched data. Table S9: EQ5D and VAS results in HCV positives by treatment duration (proxy for cirrhosis status, with cirrhosis patients treated for 24 weeks), for all participants without propensity score matching; EQ‐5D HRQoL weights calculated using Pakistan value set. Table S10: Logistic regression results for EQ5D domains between HCV‐negative and HCV‐positive after PSM. Table S11: Full logistic regression results for variables associated with problems in EQ‐5D domains among HCV‐positive participants. Table S12: EQ5D results comparing HCV‐positive and HCV‐negative groups using the UK value set (matched data). [file JVH-33-0-s001.docx]

**Supplementary materials**

**The Impact of Hepatitis C and Socio-Demographic Variables on Health-Related Quality of Life Among Patients in Pakistan**

Siwaporn Niyomsri^1^, Aaron G. Lim^1^, Ambreen Arif^2^, Muhammad Asim^3^, Auj Chaudhry^4^, Naheed Choudhry^5^, Polychronis Kemos^5^, Aliya Hasnain^6^, Noor Saba^6^, Chris Metcalfe^1^, Graham R Foster^5^, Asad Choudhry^4^, Saad Niaz^3^, Saeed Sadiq Hamid^6^, Huma Qureshi^2^, Peter Vickerman^1^, Josephine G. Walker^1^

^1^Bristol Medical School, Population Health Sciences, Bristol, United Kingdom

^2^Doctors plaza, Karachi, Pakistan

^3^Dow University of Health Sciences (DUHS), Karachi, Pakistan

^4^The Liver Clinic, Gujranwala, Pakistan

^5^Queen Mary University of London, London, United Kingdom

^6^Aga Khan University, Karachi, Pakistan

Table of Contents

**Supplementary Material 1: Screening methods 3**

**Supplementary Material 2: Estimating QALY loss due to HCV infection in Pakistan…….…….4**

Table S1: DC and HCC HRQoL taken from previously studies…………………………..…………….4

Table S2: Disutilities used in the QALY loss analyses……………….……………………………………4

Table S3: Data from a previous study - Numbers of HCV infection and deaths in 2024 from the model………………………………………………………………………………………………………………5

Table S4: GBD – DALY and QALY weight comparison……………………………………………………..5

**Supplementary Material 3: Propensity score matching (PSM) and sensitivity analysis ………………….………………………………………..……………………………………………………...….……6**

Figure S1: The distribution of propensity scores and covariance balance plot before and after propensity matching for HCV positive group and negative group…………….……………….7

Table S5: Sensitivity analyses results on matched sample……………………………………………..8

Table S6: QuEENS Checklist (TSD-17)…………………………………………………………………………8

**Supplementary Material 4: EQ5D and VAS Scores Results12**

Figure S2a: Scatter plot of EQ5D and VAS scores of all participants12

Figure S2b: Distribution of EQ5D and VAS scores 12

Table S7: EQ5D and VAS results by socio-demographic characteristics among HCV-positive and HCV-negative after propensity score matching…………………………………………………….13

Table S8: Tobit regression results showing the relationship between HCV-negative and positive among all participants using propensity-score matched data ……………………….…14

Table S9: EQ5D and VAS results in HCV-positives by treatment duration ………………..…………..……14

Table S10: Logistic regression results for EQ5D domains between HCV-negative and HCV-positive after PSM ………………………………………………………………………………….……….…….15

Table S11: Full logistic regression results for EQ5D domains among HCV-positive participants..………………………………………………………………………………………………………..16

**Supplementary Material 5: EQ5D results from the UK value set……………………………………17**

Table S12: EQ5D results comparing HCV-positive and HCV-negative groups using the UK value set (matched data)………………………………………………………………………………………..17

**References…………………………………………………………………………………………………………..18**

**Supplementary Material 1: Screening methods**

Participants were recruited through a screening process, which included both random household screenings and organised screening camps. Specifically:

1. **Household Screening:** Conducted door-to-door in rural, peri-urban, and urban settlements in District Malir, Karachi. This method ensured broad participation, with the field team making up to three attempts to contact individuals not at home initially.
2. **Screening Camps:** Organised in collaboration with local stakeholders in various Union Councils of District Malir and other locations. These camps were centrally located within walking distance of target populations to facilitate accessibility.
3. **Clinic-Based Recruitment:** Conducted in several clinics and hospitals across Karachi, including AKU liver clinic, ZMT clinics, and Dow University, among others. This involved both walk-in screenings and targeted recruitment of patients visiting for other medical services.
4. **Corporate and Factory Screenings:** Held at various corporate sites and factories, such as Soorty Mills and Ahmed Foods Factory, targeting workers for HCV testing and treatment.

**Supplementary Material 2: Estimating QALY loss due to HCV infection in Pakistan**

**Steps of calculation**

1. **Morbidity-related QALY loss**

Morbidity-related QALY loss was calculated by applying stage-specific disabilities derived from HRQoL decrements using EQ5D data. The approach incorporated the following:

- Non-cirrhosis (NC) and compensated cirrhosis (CC): Disutilities were obtained directly from EQ5D results in HCV HRQoL study.
- Decompensated cirrhosis (DC): A meta-analysis was conducted using EQ5D data reported in a systematic review and meta-analysis^1^. A fixed-effect model was applied.
- Hepatocellular carcinoma (HCC): Disutility was derived from a single study, which included HRQoL data for patients with HCC in France, the UK, and Germany^2^.

**Table S1:** DC and HCC HRQoL taken from previously studies^1,2^

| **Health stage** | **Method** | **Value** | **95% CI** | **P-value** | **I^2** |
| --- | --- | --- | --- | --- | --- |
| Decompensated cirrhosis | Fixed-effect meta-analysis | 0.645 | 0.577 – 0.713 | <0.001 | 0.73 |
| Hepatocellular carcinoma | Taken from a single study (N = 25) | 0.510 | 0.373 – 0.647 | - | - |

**Table S2:** Disutilities used in the QALY loss analyses; baseline HRQoL value for individuals without HCV was derived from the HCV-negative group, with a mean EQ5D score of 0.979 (0.977 – 0.981).

| **Health stage** | **Value** | **Lower – upper bounds** | **Reference** |
| --- | --- | --- | --- |
| Non-cirrhosis | 0.026 | 0.019 – 0.032 | HCV HRQoL study |
| Compensated cirrhosis | 0.034 | 0.017 – 0.051 | HCV HRQoL study |
| Decompensated cirrhosis | 0.334 | 0.264 – 0.404 | Buchanan-Hughes et al (2019)^1^ |
| Hepatocellular carcinoma | 0.469 | 0.330 – 0.608 | Pol et al (2015)^2^ |

For each stage, morbidity QALY loss was calculated as

$$Morbidity QALY loss=Number of population*Disutility$$

The total morbidity-related QALY loss was derived by summing the QALY losses across all disease stages. A weighted disutility for chronic HCV was calculated by determining the proportion of individuals in each disease stage and applying their respective disutilities:

$$Weighted disutility= \sum(Stage proportion*Stage disutility)$$

This weighted disutility was used to reconcile stage-specific calculations with aggregate chronic HCV population data.

1. **Mortality-related QALY loss**

To estimate the mortality-related QALY loss, we used years of life lost (YLL) data adapting a model used in a previous published study^3^. YLL accounts for the years of life lost due to premature deaths caused by HCV-related complications. For this analysis, we assumed an average life expectancy of 68 years in Pakistan, which reflects the potential lifespan lost for individuals who died prematurely for HCV-related causes. YLL was calculated based on deaths due to HCV occurring through 2024, with an estimated loss of life years based on the difference between the total lifespan of 68 and the median of the age category at time of death.

The QALY loss due to mortality was then adjusted using the HRQoL value for HCV-negative (0.979), representing the quality-adjusted life expectancy that would have been experienced in the absence of HCV.

$$Mortality related QALY loss=YLL*HCVnegative HRQoL value (0.979)$$

1. **Total QALY loss**

The total QALY loss was calculated as the sum of morbidity-and mortality-related QALY losses to reflect to overall impact of HCV infection in terms of both quality of life decrements due to morbidity across different disease stages and premature deaths caused by HCV-related complications.

$$Total QALY loss=Morbidity related QALY loss+Mortality related QALY loss$$

**Table S3:** Numbers of HCV infection, deaths, and YLLs modelled as of 2024^3^

| **Disease stage** | **Median** | **Lower bound** | **Upper bound** |
| --- | --- | --- | --- |
| Chronic HCV | 8,291,583 | 7,505,946 | 9,197,056 |
| Non cirrhosis HCV | 6,236,828 | 5,516,692 | 7,020,611 |
| Compensated cirrhosis | 1,630,781 | 1,232,040 | 2,091,389 |
| Decompensated cirrhosis | 355,855 | 193,662 | 516,132 |
| Hepatocellular carcinoma | 55,096 | 8,790 | 148,546 |
| Number of deaths | 89,300 | 62,679 | 123,114 |
| YLL | 451,768 | 317,065 | 625,140 |

**Table S4:** GBD – DALY and QALY weight comparison

| **Health stage** | **GBD 2021 – DALY weight*** | **QALY disutility** |
| --- | --- | --- |
| Chronic HCV without cirrhosis | 0.000 | 0.026 |
| Compensated cirrhosis | 0.000 | 0.034 |
| Decompensated cirrhosis | 0.178 – 0.300 | 0.334 |
| Liver cancer | 0.049 – 0.540 | 0.469 |

*Reference: GBD 2021 ^4^

**Supplementary Material 3: Propensity score matching (PSM) and sensitivity analysis**

**Propensity score matching**

A propensity score reflects the probability that a participant is HCV-positive based on observed characteristics. These scores were calculated using logistic regression (a logit model) and the resulting predicted probabilities were used to match each HCV-positive participant to HCV-negative participant with a similar score (1:1 nearest neighbour matching, using a caliper width of 0.2)^5,6^. This approach balances the two groups on observed characteristics, making the comparison more robust.

Matching success was assessed using standardised mean differences (SMDs; Table 1 in the main text), which were calculated for each variable to measure balance between groups. For categorical variables, SMDs were calculated as the difference in proportions between groups, divided by the pooled standard deviation. SMD values <0.1 indicated good balance. P-values were reported from chi-square tests (before PSM) and McNemar or Bowker tests (after PSM) to assess statistical differences in distributions.

**
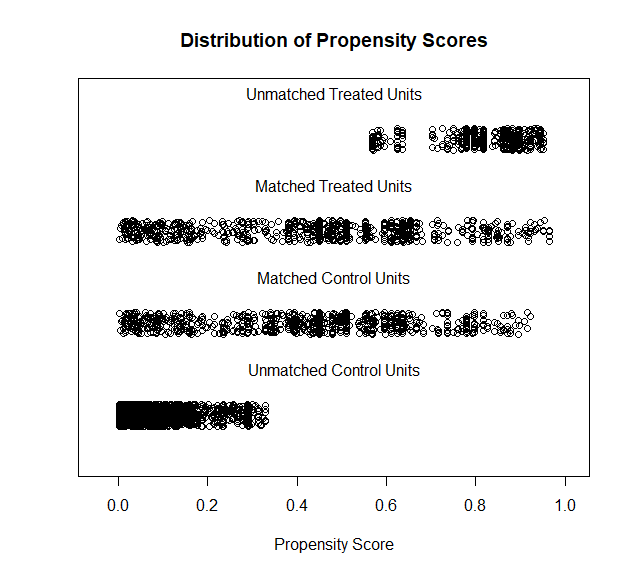

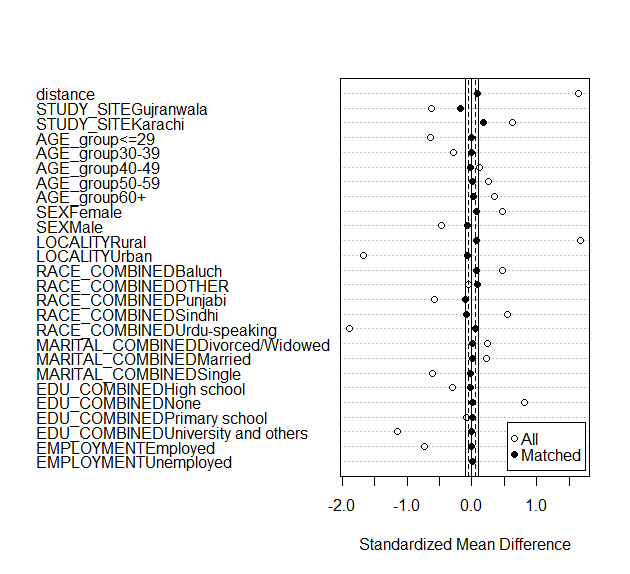
**

**Figure S1:** The distribution of propensity scores (top) and covariance balance plot before and after propensity matching for HCV positive group and negative group (bottom)

**Sensitivity analyses**

To assess whether the regression methodology was consistent and robust to alternative outcome models, we performed two sensitivity analyses. All sensitivity models were run on the PSM-matched sample (n = 1,556; 778 pairs) to target the average treatment effect on the treated (ATT). First, we fit an OLS (non-censoring) of EQ-5D on HCV status, adjusting for the PSM covariates to control any residual bias. Then, we fit a logistic regression for non-perfect health (EQ-5D < 1), which directly targets the ceiling estimand. We report odds ratio (OR) and average marginal risk differences (RD, percentage points) via marginal standardization. We also applied the QuEENS checklist (Quality of Effectiveness from Non-randomised Studies) to evaluate general and PSM-specific method of study quality.

**Table S5:** Sensitivity analyses results on matched sample

| **Outcome** | **Model** | **Results** | **Estimate** | **95% CI** | **P-value** | **N** |
| --- | --- | --- | --- | --- | --- | --- |
| EQ-5D utility (continuous) | OLS (non-censoring) | Adjusted mean difference | -0.012 | -0.019, -0.006 | <0.001 | 1,556 |
| Any problem  (EQ-5D < 1) | Logistic | Risk difference (pp), odds ratio | 19.4 pp, OR = 2.20 | 13.7 – 25.1 pp; OR 95% CI 1.74 – 2.79 | <0.001 | 1,556 |

**Abbreviations:** OLS, ordinary least squares; OR, odds ratio; pp, percentage points; 95% CI, 95% confidence interval.

For sensitivity regression on the matched data, OLS (non-censoring) estimated an adjusted mean difference in EQ-5D of -0.012 (95% CI -0.019 to -0.006). For EQ-5D < 1, the logistic model indicated that HCV-positive participants had 19.4 percentage points higher risk of any problem (95% CI 13.7 – 2.51 pp) with OR = 2.20 (95% CI 1.74 – 2.79). These results are consistent with the primary Tobit analysis on the matched data. Moreover, because most values below 1 cluster at 0.95 – 0.99, yielding a small mean OLS gap (-0.012) and Tobit (AME = -0.018), indicating a shift from perfect to near-perfect health rather than large drops below 1.

**Table S6:** QuEENS Checklist (TSD-17)^7^

| Item | QuEENS item | Questions | Status | Comments |
| --- | --- | --- | --- | --- |
| General issues | | | | |
| 1 | Compare methods | Have different methods been compared within the study? | [X] Yes  [ ] Partial  [ ] No  [ ] N/A | Primary analysis uses PSM and Tobit for EQ-5D domain differences via logistic regression. In the sensitivity analysis, we add OLS for non-censoring on EQ-5D and logistic (EQ-5D < 1) on any problem. Conclusions are consistent in direction. |
| 2 | Compare to external literature | Have the results of the study been compared to others in the literature? | [ ] Yes, compared to alternative methods using the same dataset  [X] Yes, compared to similar methods using other data sources  [ ] Not compared – no other estimates found in the literature  [ ] Not compared  [ ] N/A | We reference EQ-5D reports in other conditions with similar methods (Tobit) or compared with other regressions in published studies. For HCV comparison, we mention sources for HRQoL inputs in QALY-burden and stage disutilities. |
| 3 | Estimand & assumptions | Is there a discussion of what treatment effects is identified and of the assumption needed? | [X] Discussion of effect and assumptions  [ ] Discussion of effect but not the assumptions  [ ] Discussion of the assumptions but not the effect  [ ] No discussion of either  [ ] N/A | All regression models used ATT on the matched sample. The OLS and Tobit estimate mean differences and logistic reported as RD/OR for EQ-5D < 1. |
| 4 | Outcome model suitability & diagnostics | Is the model chosen consistent with the outcome variable if using a parametric method? | [X] Yes  [ ] Unclear  [ ] No  [ ] N/A | Tobit regression was chosen due to bounded EQ-5D score with large spike at 1. Logistic models used for address the ceiling directly. The OLS to test robustness while ignoring censoring for robustness check. |
| 5 | Specification checks & robustness | Were any checks conducted on the model specification? | [X] Yes, appropriate (detail which)  [ ] Yes, but inappropriate or not enough  [ ] No checks reported  [ ] N/A | Robustness shown across model families. Tobit vs logistic (domains) and new OLS/non-ceiling logistic. Non-parametric tests are also reported. We used robust SE by matched pair and marginal standardization for risk differences. |
| Methods assuming selection on observables | | | | |
| 6 | Selection-on-observables justification | On selection: Is the assumption of selection on observables assessed? | [ ] Yes, expert literature or opinion cited  [X] Yes, theoretical reasoning given  [ ] No  [ ] N/A | Covariates reflect socio-demographic confounding from  two-gate recruitment. All are pre-treatment. |
| 7 | Overlap/  positivity | What checks were conducted to assess overlap? | [X] Yes, thorough checks  [ ] Yes, minimum checks  [ ] No checks reported  [ ] N/A | Overlap shown via propensity-score distribution and balance plots. Post match SMD < 0.1. |
| 8 | Post-match balance | Has balancing of the covariates been checked after matching and propensity score methods? | [X] Yes, thorough checks  [ ] Yes, minimum checks  [ ] No checks reported  [ ] N/A | We report before and after SMDs and display PS and balance plots. |
| Methods using the propensity score | | | | |
| 9 | Propensity score flexibility | Is the propensity score function sufficiently flexible? | [X] Yes, includes interactions or different functions of the covariates  [ ] Yes, flexible due to the semi-parametric specification  [ ] Unlikely to be flexible enough  [ ] Unclear or not reported  [ ] N/A | Propensity score estimated via logit with categorical variables (with age in bands). We tested 2-way interactions (age * sex, screening site * residential area). After 1:1 nearest-neighbor matching (caliper 0.2), all adjusted SMD < 0.1 for both the base and interaction specs. Treatment-effect estimates were unchanged in direction and magnitude. |
| 10 | Covariate inclusion/  exclusion rules | Are potential IVs excluded from the set of conditioning variables? | [X] Yes  [ ] Some variables might present a problem  [ ] IV clearly included  [ ] N/A | We include only pre-treatment socio-demographics. No post-treatment variables or instruments. Avoiding colliders. |
| Matching methods | | | | |
| 11 | Data quality and harmonization | Data quality: Are these data quality issues? | [X] Data and definitions comparable for treated and control groups - [X] Yes, [ ] No, [ ] Unclear or not reported  [X] Treated and controls come from the same area or environment – [X] Yes, [ ] No, [ ] unclear or not reported  [X] Rich set of variables used for matching – [X] Yes, available and used, [ ] Not available or not used  [X] Reasonable sample sizes – [X] Yes, likely, [ ] No  [ ] N/A | Recruitment sources, EQ5D-3L in Urdu with Pakistani value set and data summarization methods are documented in the study protocol. Definitions are consistent across groups. |
| 12 | Bias-adjustment after matching | For Nearest Neighbor: Has bias adjustment been conducted if more than one variable was included when matching on covariates? | [X] Yes  [ ] No  [ ] N/A | We perform multivariable Tobit on the matched sample for residual bias and precision. |
| 13 | Matching with/without replacement | Is the choice of replacement (with/without) reasonable? | [X] Yes  [ ] Likely  [ ] No  [ ] N/A | 1:1 nearest-neighbor with caliper = 0.2. Matching was without replacement. |
| 14 | Caliper/ratio choice & sensitivity | Is the choice of the number of matches/caliper matching/radius matching reasonable? | [X] Yes  [ ] Likely  [ ] No  [ ] N/A | We report caliper 0.2 and 1:1 ratio, which is the optimal option. Balance results changed under small caliper (0.1) and higher caliper (0.25). |
| IV methods | | | | |
| 15 | IV relevance | Is the instrument well justified? (i.e. eligibility in program participation (reason), natural experiment, theoretically sensible, fitted propensity scores). | [ ] Yes  [ ] Partial  [ ] No  [X] N/A |  |
| 16 | IV exclusion restriction | Is the sample size relatively large? | [ ] Yes  [ ] No  [X] N/A |  |
| 17 | IV independence/  balance | If more than one IV, is the test of over-identifying restrictions reported? | [ ] Yes  [ ] No  [X] N/A |  |
| 18 | IV monotonicity/  LATE | Is a weak instrument(s) test reported? | [ ] Yes  [ ] No  [X] N/A |  |
| Difference in Differences (standard) | | | | |
| 19 | DiD: parallel trends | Does the intervention generate exogenous variation? (not application if natural experiment) | [ ] Yes, highly likely  [ ] Unlikely  [X] N/A |  |
| 20 | DiD: no anticipation & stable composition | Is the assumption of common trends across groups reasonable? | [ ] Yes, highly likely  [ ] Unlikely  [X] N/A |  |
| 21 | DiD: timing/staggered adoption | Is it reasonable to assume that there is no selection of unobserved temporary individual specific shocks? | [ ] Yes, highly likely  [ ] No, unlikely  [X] N/A |  |
| 22 | DiD: dynamic effects | Is the assumption of no systematic composition changes within each group reasonable? (applicable with repeated cross-sections, not with longitudinal data) | [ ] Yes, highly likely  [ ] Unlikely  [X] N/A |  |
| Regression discontinuity design | | | | |
| 23 | RDD: bandwidth & sample size | Is the sample size relatively large? | [ ] Yes  [ ] No  [X] N/A |  |
| 24 | RDD: manipulation & continuity tests | Is the assumption that individuals are not able to affect the instrument to change the likelihood of participation reasonable? | [ ] Yes, highly likely  [ ] Unlikely  [X] N/A |  |

**Supplementary Material 4: EQ5D and VAS Scores Results**


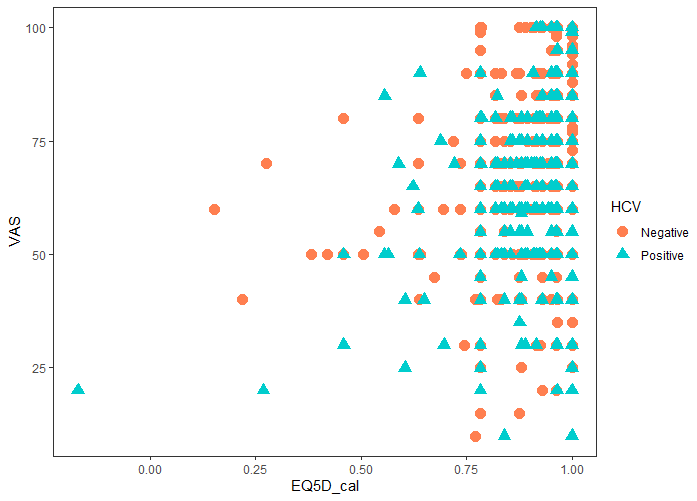


**Figure S2a:** Scatter plot of EQ5D and VAS scores of all participants


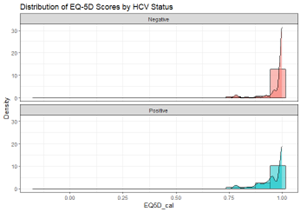

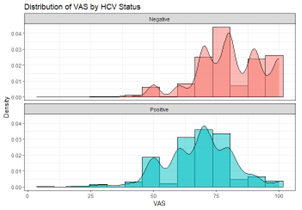


**Figure S2b:** Distribution of EQ5D and VAS scores of all participants

**Table S7:** EQ5D and VAS results by socio-demographic characteristics among HCV-positive and HCV-negative after propensity score matching

| **Variables/outcome** | **EQ-5D score** | | **VAS score** | |
| --- | --- | --- | --- | --- |
|  | **HCV-negative**  **Mean (95% CI)** | **HCV-positive**  **Mean (95% CI)** | **HCV-negative**  **Mean (95% CI)** | **HCV-positive**  **Mean (95% CI)** |
| Gender  Male  Female | 0.985 (0.981 – 0.989)  0.957 (0.949 – 0.966) | 0.967 (0.959 – 0.975)  0.954 (0.948 – 0.960) | 78.60 (77.26 – 79.93)  73.38 (72.01 – 74.75) | 71.67 (70.01 – 73.33)  68.21 (67.00 – 69.42) |
| Age  ≤ 29  30 – 39  40 – 49  50 - 59  60 and over | 0.990 (0.983 – 0.996)  0.984 (0.977 – 0.991)  0.969 (0.958 – 0.979)  0.961 (0.949 – 0.973)  0.940 (0.922 – 0.958) | 0.980 (0.971 – 0.989)  0.969 (0.959 – 0.980)  0.954 (0.944 – 0.964)  0.958 (0.948 – 0.968)  0.933 (0.917 – 0.948) | 79.24 (77.12 – 81.36)  78.49 (76.68 – 80.30)  75.47 (73.56 – 77.38)  74.45 (72.16 – 76.74)  70.04 (67.16 – 72.92) | 71.08 (68.76 – 73.39)  70.00 (67.94 – 72.06)  69.93 (67.93 – 71.92)  70.25 (67.81 – 72.69)  66.81 (64.28 – 69.34) |
| Occupation  Employed  Unemployed | 0.983 (0.979 – 0.987)  0.954 (0.944 – 0.964) | 0.967 (0.962 – 0.973)  0.950 (0.941 – 0.959) | 77.42 (76.28 – 78.56)  73.85 (72.18 – 75.52) | 71.74 (70.42 – 73.05)  67.12 (65.60 – 68.64) |
| Marital status  Single  Married  Divorced/widowed | 0.986 (0.977 – 0.995)  0.973 (0.968 – 0.978)  0.904 (0.868 – 0.940) | 0.952 (0.926 – 0.979)  0.963 (0.958 – 0.968)  0.916 (0.890 – 0.943) | 79.93 (77.10 – 82.76)  76.03 (75.01 – 77.04)  65.92 (59.42 – 72.35) | 67.42 (63.89 – 70.96)  70.33 (69.25 – 71.41)  64.13 (59.97 – 68.28) |
| Residential area  Urban  Rural | 0.953 (0.941 – 0.966)  0.978 (0.973 – 0.982) | 0.956 (0.945 – 0.967)  0.961 (0.955 – 0.967) | 75.99 (74.09 – 77.89)  75.87 (74.73 – 77.00) | 73.68 (71.40 – 75.95)  68.41 (67.33 – 69.50) |
| Ethnicity  Punjabi  Sindhi  Baluch  Urdu-speaking  Other | 0.914 (0.893 – 0.935)  0.985 (0.980 – 0.989)  0.980 (0.971 – 0.989)  0.990 (0.979 – 1.000)  0.969 (0.957 – 0.980) | 0.932 (0.915 – 0.949)  0.965 (0.958 – 0.972)  0.951 (0.938 – 0.963)  0.986 (0.977 – 0.996)  0.971 (0.962 – 0.980) | 74.57 (70.85 – 78.29)  76.19 (75.02 – 77.36)  76.73 (74.41 – 79.05)  79.42 (75.29 – 83.56)  74.50 (72.24 – 76.77) | 69.34 (66.37 – 70.65)  70.34 (68.99 -71.68)  68.51 (66.37 – 70.65)  77.19 (71.77 – 82.60)  68.31 (65.60 – 71.02) |
| Education level  No education  Primary school  Secondary school  University and others | 0.963 (0.955 – 0.971)  0.978 (0.970 – 0.986)  0.980 (0.971 – 0.989)  0.984 (0.973 – 0.995) | 0.952 (0.945 – 0.960)  0.963 (0.952 – 0.974)  0.972 (0.963 – 0.981)  0.978 (0.967 – 0.990) | 73.66 (72.38 – 74.94)  79.02 (76.87 – 81.17)  79.08 (76.91 – 81.26)  76.95 (72.79 – 81.12) | 68.26 (66.97 – 69.55)  71.15 (68.94 – 73.35)  71.80 (69.11 – 74.50)  72.58 (68.57 – 76.59) |
| Study site  Karachi  Gujranwala | 0.981 (0.978 – 0.985)  0.895 (0.870 – 0.920) | 0.964 (0.959 – 0.969)  0.917 (0.895 – 0.940) | 76.21 (75.30 – 77.13)  73.68 (69.19 – 78.16) | 69.74 (68.71 – 70.76)  70.08 (65.76 – 74.39) |
| Overall EQ-5D/VAS score | 0.971 (0.966 – 0.976) | 0.960 (0.955 – 0.965) | 75.90 (74.93 – 76.87) | 69.77 (68.76 – 70.77) |

**Abbreviations:** HCV, hepatitis C virus; 95% CI, 95% confidence interval; EQ5D, European Quality of Life 5 Dimensions; VAS, Visual Analog Scale.

**Table S8:** Tobit regression results showing the relationship between HCV-negative and positive among all participants using propensity-score matched data

| **Parameters** | **B** | **SE** | **95% CI** | **p-value** |
| --- | --- | --- | --- | --- |
| Intercept | 1.05 | 0.02 | 1.01, 1.10 | **<0.001** |
| HCV | -0.05 | 0.01 | -0.07, -0.04 | **<0.001** |
| Age [ref: ≤ 29]  30 – 39  40 – 49  50 - 59  60 and over | -0.05  -0.09  -0.10  -0.13 | 0.02  0.02  0.02  0.02 | -0.08, -0.01  -0.12, -0.05  -0.13, -0.06  -0.17, -0.10 | **0.005**  **<0.001**  **<0.001**  **<0.001** |
| Study site [ref: Gujranwala]  Karachi | 0.11 | 0.03 | 0.07, 0.16 | **<0.001** |
| Gender [ref: female]  Male | 0.04 | 0.01 | 0.02, 0.06 | **<0.001** |
| Residential area [ref: rural]  Urban | 0.01 | 0.01 | -0.02, 0.03 | 0.597 |
| Employment status [ref: employed]  Unemployed | -0.03 | 0.01 | -0.05, -0.01 | **0.001** |
| Marital status [ref: married]  Single  Divorced or widowed | -0.04  -0.04 | 0.02  0.02 | -0.07, -0.01  -0.08, -0.01 | **0.023**  **0.021** |
| Ethnicity [ref: Punjabi]  Urdu-speaking  Baluch  Sindhi  Other | 0.08  -0.01  0.02  0.01 | 0.03  0.03  0.02  0.02 | 0.01, 0.15  -0.06, 0.04  -0.03, 0.06  -0.04, 0.05 | **0.018**  0.682  0.461  0.882 |
| Education level [ref: primary school]  No education  Secondary school  University and others | -0.01  0.01  0.02 | 0.01  0.02  0.02 | -0.03, 0.02  -0.02, 0.04  -0.03, 0.05 | 0.667  0.390  0.469 |
| LogSigma | -1.98 | 0.04 | -2.04, -1.91 | **<0.001** |

**Abbreviations:** B, beta estimates; SE, standard error; 95%CI, 95% confidence interval; ref, reference.

**Table S9:** EQ5D and VAS results in HCV-positives by treatment duration (proxy for cirrhosis status, with cirrhosis patients treated for 24 weeks), for all participants without propensity score matching; EQ-5D HRQoL weights calculated using Pakistan value set^8^

| **Health status** | **Total** | **Mean** | **Median** | **Min - Max** | **SD** | **95% CI** | **P-value** |
| --- | --- | --- | --- | --- | --- | --- | --- |
| **EQ-5D HRQoL**  HCV Positive  12-week treatment  24-week treatment | 1,263  915  206 | 0.952  0.953  0.945 | 1.000  1.000  0.965 | -0.171 – 1.000  0.268 – 1.000  -0.171 – 1.000 | 0.081  0.077  0.107 | 0.947 – 0.956  0.949 – 0.958  0.930 – 0.960 | 0.112* |
| **VAS**  HCV Positive  12-week treatment  24-week treatment | 1,263  915  206 | 67.86  68.15  65.85 | 70.00  70.00  70.00 | 10.00 – 100.00  10.00 – 100.00  10.00 – 100.00 | 13.87  13.72  15.70 | 67.10 – 68.63  67.26 – 69.04  63.69 – 68.01 | 0.060* |

*p-value between 12 and 24-week treatment

**Abbreviations:** EQ5D, European Quality of Life 5 Dimensions; VAS, Visual Analog Scale; HCV, hepatitis C virus; SD, standard deviation; CI, confidence interval; HRQoL, health-related quality of life

**HCV Positive:** Participants with positive results for both anti-HCV antibody and HCV RNA tests.

**Table S10:** Logistic regression results for EQ5D domains between HCV-negative and HCV-positive after PSM

| **Independent variables** | **Mobility difficulties** | | **Usual activities difficulties** | | **Pain/discomfort** | | **Self-care difficulties** | | **Anxiety/depression** | |
| --- | --- | --- | --- | --- | --- | --- | --- | --- | --- | --- |
|  | **OR (95% CI)** | **p** | **OR (95% CI)** | **p** | **OR (95% CI)** | **p** | **OR (95% CI)** | **p** | **OR (95% CI)** | **p** |
| Intercept | 0.19 (0.09 – 0.41) | <0.001 | 0.08 (0.03 – 0.24) | <0.001 | 0.63 (0.29 – 1.36) | 0.238 | 0.09 (0.03 – 0.31) | <0.001 | 0.16 (0.08 – 0.34) | <0.001 |
| HCV positive | 2.38 (1.80 – 3.16) | <0.001 | 1.53 (1.04 – 2.25) | 0.030 | 1.69 (1.25 – 2.28) | 0.001 | 2.37 (1.54 – 3.66) | <0.001 | 1.32 (1.00 – 1.75) | 0.048 |
| Study site  [ref: Gujranwala]  Karachi | 0.37 (0.17 – 0.80) | 0.012 | 0.21 (0.08 – 0.56) | 0.002 | 0.10 (0.05 – 0.23) | <0.001 | 0.14 (0.04 – 0.47) | 0.001 | 0.51 (0.26 – 1.00) | 0.050 |
| Age  [ref: ≤ 29]  30 – 39  40 – 49  50 – 59  60 and over | 1.51 (0.86 – 2.65)  2.73 (1.60 – 4.67)  4.25 (2.42 – 7.45)  7.92 (4.39 – 14.27) | 0.148  <0.001  <0.001  <0.001 | 2.54 (0.97 – 6.64)  3.90(1.55 – 9.86)  4.77 (1.82 – 12.48)  9.71 (3.68 – 25.61) | 0.057  0.004  0.001  <0.001 | 1.40 (0.79 – 2.51)  2.35 (1.35 – 4.08)  2.52 (1.39 – 4.55)  3.51 (1.88 – 6.53) | 0.253  0.003  0.002  <0.001 | 1.80 (0.61 – 5.34)  4.43 (1.64 – 11.97)  4.40 (1.55 – 12.49)  9.30 (3.23 – 26.74) | 0.287  0.003  0.005  <0.001 | 1.63 (0.94 – 2.83)  2.70 (1.59 – 4.60)  2.29 (1.30 – 4.05)  1.87 (1.01 – 3.48) | 0.085  <0.001  0.004  0.048 |
| Gender  [ref: female]  Male | 0.38 (0.27 – 0.52) | <0.001 | 0.51 (0.33 – 0.78) | 0.002 | 0.59 (0.42 – 0.83) | 0.002 | 0.36 (0.22 – 0.59) | <0.001 | 0.92 (0.67 – 1.26) | 0.592 |
| Residential areas  [ref: rural]  Urban | 0.60 (0.41 – 0.89) | 0.011 | 1.15 (0.70 – 1.89) | 0.585 | 0.95 (0.63 – 1.42) | 0.784 | 0.66 (0.38 – 1.15) | 0.143 | 1.17 (0.81 – 1.67) | 0.406 |
| Ethnicity  [ref: Punjabi]  Urdu-speaking  Baluch  Sindhi  Other | 0.54 (0.19 – 1.59)  1.24 (0.57 – 2.71)  0.93 (0.44 – 1.98)  1.03 (0.47 – 2.27) | 0.265  0.592  0.849  0.941 | 0.13 (0.02 – 1.10)  0.98 (0.35 – 2.73)  0.76 (0.28 – 2.06)  0.50 (0.17 – 1.46) | 0.061  0.961  0.583  0.205 | 0.12 (0.03 – 0.57)  1.06 (0.47 – 2.39)  0.85 (0.39 – 1.85)  0.82 (0.36 – 1.84) | 0.008  0.884  0.673  0.622 | 0.16 (0.02 – 1.45)  0.97 (0.29 – 3.31)  0.72 (0.22 – 2.38)  0.71 (0.20 – 2.55) | 0.103  0.963  0.584  0.598 | 0.22 (0.07 – 0.65)  0.78 (0.39 – 1.56)  0.52 (0.27 – 1.00)  0.73 (0.37 – 1.44) | 0.006  0.482  0.051  0.368 |
| Marital status  [ref: married]  Single  Divorced or widowed | 1.22 (0.68 – 2.17)  1.00 (0.58 – 1.73) | 0.506  0.993 | 2.29 (1.05 – 4.99)  1.18 (0.61 – 2.26) | 0.037  0.621 | 1.75 (0.99 – 3.12)  1.99 (1.11 – 3.56) | 0.055  0.021 | 2.21 (0.97 – 5.06)  1.74 (0.90 – 3.38) | 0.060  0.100 | 1.27 (0.73 – 2.21)  2.76 (1.62 – 4.71) | 0.406  <0.001 |
| Education levels  [ref: primary school]  No education  High school  University and others | 0.90 (0.61 – 1.31)  0.96 (0.59 – 1.58)  0.73 (0.37 – 1.47) | 0.568  0.884  0.380 | 1.19 (0.71 – 2.02)  1.03 (0.51 – 2.08)  0.29 (0.07 – 1.30) | 0.506  0.929  0.106 | 1.08 (0.72 – 1.62)  0.88 (0.52 – 1.49)  0.69 (0.33 – 1.45) | 0.710  0.624  0.323 | 1.34 (0.74 – 2.45)  1.14 (0.51 – 2.55)  1.30 (0.44 – 3.87) | 0.334  0.741  0.639 | 1.23 (0.84 – 1.80)  0.91 (0.55 – 1.50)  1.49 (0.83 – 2.68) | 0.288  0.715  0.187 |
| Employment  [ref: employed]  Unemployed | 1.50 (1.10 – 2.03) | 0.010 | 1.64 (1.08 – 2.49) | 0.019 | 1.36 (0.99 – 1.88) | 0.060 | 1.06 (0.67 – 1.68) | 0.809 | 1.69 (1.24 – 2.30) | 0.001 |

**Abbreviations:** HCV, hepatitis C virus; OR, odds ratios; p, p-value; 95% CI, 95% confident interval; ref, reference.

**Reference categories:** age, ≤ 29; study site, Gujranwala; gender, female; residential area, rural; employment status, currently employed; marital status, married; ethnicity, Punjabi; education level, primary school.

| **Independent variables** | **Mobility difficulties** | | **Usual activities difficulties** | | **Pain/discomfort** | | **Self-care difficulties** | | **Anxiety/depression** | |
| --- | --- | --- | --- | --- | --- | --- | --- | --- | --- | --- |
|  | **OR (95% CI)** | **p** | **OR (95% CI)** | **p** | **OR (95% CI)** | **p** | **OR (95% CI)** | **p** | **OR (95% CI)** | **p** |
| Intercept | 0.36 (0.12 – 1.07) | 0.067 | 0.04 (0.01 – 0.27) | 0.001 | 1.17 (0.38 – 3.59) | 0.788 | 0.09 (0.01 – 0.53) | 0.008 | 0.24 (0.08 – 0.69) | 0.008 |
| Study site  [ref: Gujranwala]  Karachi | 0.55 (0.19 – 1.57) | 0.267 | 0.27 (0.08 – 0.90) | 0.032 | 0.08 (0.03 – 0.25) | <0.001 | 0.21 (0.05 – 0.87) | 0.031 | 0.72 (0.27 – 1.88) | 0.498 |
| Age  [ref: ≤ 29]  30 – 39  40 – 49  50 – 59  60 and over | 2.07 (1.06 – 4.03)  2.85 (1.51 – 5.37)  3.14 (1.63 – 6.04)  7.25 (3.73 – 14.07) | 0.033  0.001  <0.001  <0.001 | 6.23 (1.38 – 28.05)  7.18 (1.64 – 31.46)  9.17 (2.06 – 40.78)  16.80 (3.80 – 74.29) | 0.017  0.009  0.004  <0.001 | 1.59 (0.80 – 3.14)  1.80 (0.94 – 3.45)  2.23 (1.15 – 4.35)  2.85 (1.45 – 5.59) | 0.184  0.076  0.018  0.002 | 6.21 (1.37 – 28.03)  6.81 (1.56 – 29.80)  11.93 (2.69 – 52.83)  18.08 (4.07 – 80.30) | 0.018  0.011  0.001  <0.001 | 1.77 (0.90 – 3.49)  2.16 (1.13 – 4.15)  2.16 (1.10 – 4.24)  2.31 (1.16 – 4.61) | 0.099  0.020  0.026  0.018 |
| Gender  [ref: female]  Male | 0.49 (0.35 – 0.68) | <0.001 | 0.92 (0.59 – 1.43) | 0.710 | 1.04 (0.74 – 1.47) | 0.820 | 0.44 (0.28 – 0.71) | 0.001 | 1.09 (0.77 – 1.54) | 0.640 |
| Residential areas  [ref: rural]  Urban | 0.42 (0.24 – 0.73) | 0.002 | 0.94 (0.45 – 1.93) | 0.856 | 0.75 (0.43 – 1.32) | 0.321 | 1.55 (0.24 – 1.23) | 0.145 | 1.10 (0.68 – 1.79) | 0.704 |
| Ethnicity  [ref: Punjabi]  Urdu-speaking  Baluch  Sindhi  Other | 0.42 (0.11 – 1.56)  1.01 (0.46 – 2.24)  0.81 (0.37 – 1.76)  0.71 (0.31 – 1.66) | 0.193  0.972  0.591  0.432 | 0.22 (0.03 – 1.97)  0.94 (0.34 – 2.58)  0.67 (0.25 – 1.82)  0.35 (0.11 – 1.11) | 0.178  0.904  0.435  0.073 | 0.09 (0.01 – 0.79)  1.16 (0.51 – 2.67)  0.78 (0.35 – 1.77)  0.65 (0.27 – 1.57) | 0.029  0.722  0.557  0.337 | 0.32 (0.03 – 3.11)  1.32 (0.39 – 4.46)  0.97 (0.29 – 3.25)  0.65 (0.17 – 2.47) | 0.325  0.660  0.963  0.531 | 0.18 (0.05 – 0.69)  0.59 (0.28 – 1.22)  0.48 (0.24 – 0.99)  0.53 (0.25 – 1.15) | 0.013  0.155  0.046  0.110 |
| Marital status  [ref: married]  Single  Divorced or widowed | 0.91 (0.44 – 1.88)  1.52 (0.95 – 2.43) | 0.798  0.084 | 2.46 (0.98 – 6.21)  1.04 (0.58 – 1.86) | 0.056  0.907 | 1.48 (0.73 – 3.02)  1.20 (0.73 – 1.97) | 0.276  0.474 | 2.91 (1.20 – 7.06)  1.25 (0.71 – 2.19) | 0.018  0.436 | 1.18 (0.58 – 2.39)  2.01 (1.24 – 3.26) | 0.648  0.005 |
| Education levels  [ref: primary school]  No education  High school  University and others | 0.92 (0.62 – 1.38)  0.69 (0.39 – 1.23)  0.64 (0.26 – 1.58) | 0.696  0.210  0.333 | 1.30 (0.72 – 2.34)  0.86 (0.36 – 2.02)  0.27 (0.03 – 2.13) | 0.384  0.723  0.212 | 1.10 (0.71 – 1.69)  0.64 (0.33 – 1.22)  0.71 (0.27 – 1.84) | 0.685  0.171  0.479 | 1.13 (0.62 – 2.05)  0.57 (0.22 – 1.49)  0.94 (0.25 – 3.60) | 0.694  0.251  0.933 | 0.89 (0.58 – 1.35)  0.75 (0.41 – 1.36)  1.41 (0.66 – 2.99) | 0.570  0.344  0.371 |
| Employment  [ref: employed]  Unemployed | 1.36 (1.00 – 1.85) | 0.050 | 1.91 (1.22 – 2.99) | 0.005 | 1.90 (1.34 – 2.68) | <0.001 | 1.08 (0.71 – 1.64) | 0.727 | 1.70 (1.20 – 2.39) | 0.003 |
| Treatment duration  [ref: 12 week]  24-week | 1.27 (0.90 – 1.80) | 0.181 | 0.95 (0.58 – 1.55) | 0.842 | 1.27 (0.88 – 1.84) | 0.199 | 0.79 (0.48 – 1.31) | 0.358 | 1.09 (0.75 – 1.59) | 0.639 |

**Table S11:** Full logistic regression results for variables associated with problems in EQ-5D domains among HCV-positive participants

**Abbreviations:** HCV, hepatitis C virus; OR, odds ratios; p, p-value; 95% CI, 95% confident interval; ref, reference.

**Supplementary Material 5: EQ5D results from the UK value set^9^**

**Table S12:** EQ5D results comparing HCV-positive and HCV-negative groups using the UK value set (matched data)

| **Health status** | **Total** | **Mean** | **Median** | **Min - Max** | **SD** | **95% CI** | **P-value*** |
| --- | --- | --- | --- | --- | --- | --- | --- |
| **EQ5D**  HCV Positive  HCV Negative | 778  778 | 0.888  0.920 | 1.000  1.000 | -0.594 – 1.000  -0.239 – 1.000 | 0.170  0.161 | 0.876 – 0.900  0.908 – 0.931 | <0.001* |

*p-value between HCV Positive and HCV negative from Mann-Whitney U test

**Abbreviations:** EQ5D, European Quality of Life 5 Dimensions; HCV, hepatitis C virus; SD, standard deviation; CI, confidence interval; PSM, propensity score matching.

**HCV Positive:** Participants with positive results for both anti-HCV antibody and HCV RNA tests.

**HCV Negative:** Participants with negative result for anti-HCV antibody.

**References**

1. Buchanan-Hughes A.M., Buti M, Hanman K, Langford B, Wright M, Eddowes LA. Health state utility values measured using the EuroQol 5-dimensions questionnaire in adults with chronic hepatitis C: a systematic literature review and meta-analysis. *Qual Life Res* 2019; **28**(2): 297-319.

2. Pol S, Chevalier J, Branchoux S, Perry R, Miligan G, Gaudin A-F. P0747: Health related quality of life and utility values in chronic hepatitis C patients: A cross-sectional study in France, the Uk and Germany. Journal of Hepatology 2015.

3. Lim AG, Scott N, Walker JG, Hamid S, Hellard M, Vickerman P. Health and economic benefits of achieving hepatitis C virus elimination in Pakistan: A modelling study and economic analysis. *PLoS Med* 2021; **18**(10): e1003818.

4. Diseases GBD, Injuries C. Global incidence, prevalence, years lived with disability (YLDs), disability-adjusted life-years (DALYs), and healthy life expectancy (HALE) for 371 diseases and injuries in 204 countries and territories and 811 subnational locations, 1990-2021: a systematic analysis for the Global Burden of Disease Study 2021. *Lancet* 2024; **403**(10440): 2133-61.

5. Austin PC. Optimal caliper widths for propensity-score matching when estimating differences in means and differences in proportions in observational studies. *Pharm Stat* 2011; **10**(2): 150-61.

6. Austin PC. A comparison of 12 algorithms for matching on the propensity score. *Stat Med* 2014; **33**(6): 1057-69.

7. Faria R, Hernandez Alava M, Manca A, Wailoo AJ. NICE DSU Technical Support Document 17: The use of observational data to inform estimates of treatment effectiveness for Technology Appraisal: Methods for comparative individual patient data; 2015.

8. Malik M, Gu NY, Hussain A, Roudijk B, Purba FD. The EQ-5D-3L Valuation Study in Pakistan. *Pharmacoecon Open* 2023; **7**(6): 963-74.

9. Dolan P. Modeling valuations for EuroQol health states. *Med Care* 1997; **35**(11): 1095-108.
